# Safety and Effectiveness of Insulin Degludec in Patients With Type 2 Diabetes Mellitus With Chronic Liver Disease

**DOI:** 10.7759/cureus.102882

**Published:** 2026-02-03

**Authors:** Vipul Gupta, Girish Khurana

**Affiliations:** 1 Medicine, Gupta Ultrasound and Heart Care Centre, New Delhi, IND; 2 Medicine, Vidya Medical Centre, Bahadurgarh, IND

**Keywords:** basal insulin analog, insulin degludec, liver disease, long-acting insulin, type 2 diabetes mellitus

## Abstract

Background

Managing patients with type 2 diabetes mellitus (T2DM) with chronic liver disease (CLD) is challenging due to an increased risk of hypoglycemia. Among other long-acting basal insulin analogs, insulin degludec has been shown to have a low risk of hypoglycemic events in patients with T2DM.

Aim

To evaluate the effectiveness of insulin degludec in managing T2DM in patients with CLD based on changes in glycated hemoglobin (HbA1c), fasting plasma glucose, and postprandial glucose (PPG) levels, and to evaluate its safety, based on the incidence of hypoglycemic events.

Methods

This single-center, observational, retrospective study was conducted using data from 35 patients, aged between 18 and 75 years, with T2DM, a body mass index (BMI) of <40 kg/m^2^,and stable hepatic impairment based on the Child-Pugh classification. Data, including demographics, laboratory results, and medical history, were collected from electronic medical records at baseline and three months after treatment with insulin degludec. The primary endpoints were the number and severity of hypoglycemic events, as well as changes in glycated hemoglobin (HbA_1__c.)_, fasting plasma glucose (FPG), and postprandial glucose (PPG) levels. A p-value of <0.05 was considered statistically significant.

Results

Among the 35 patients enrolled in the study, the majority (n=25) were males. Most of the patients had been living with T2DM for a mean duration of 10.79±5.63 years and had mild-to-moderate hepatic impairment based on Child-Pugh scores. Most of the patients (15, 42.9%) were on a combination of sulfonylurea and insulin at baseline. Significant reductions in glycemic parameters were observed from baseline to three months after treatment (p<0.001). About 14.3% of patients developed level 1 hypoglycemia, another 14.3% developed nocturnal hypoglycemia, and none reported level 2 or 3 hypoglycemia.

Conclusion

Insulin degludec improved glycemic parameters while reducing the risk of severe hypoglycemic events. The study findings suggest that insulin degludec can be considered a safe option for patients with T2DM with CLD. However, prospective studies with larger sample sizes and a comparator arm are warranted to highlight insulin degludec’s potential in this patient population.

## Introduction

According to the Global Burden of Disease Study 2021, diabetes was estimated to have affected 529 million people worldwide. The number of patients with diabetes is estimated to exceed 1.31 billion by 2050 [1]. The World Health Organization has reported that in India, 77 million people over 18 years of age have type 2 diabetes mellitus (T2DM), and about 25 million are prediabetic [2]. Between 1991 and 2021, India observed a whopping 262.9% increase in the number of disability-adjusted life-years among patients with diabetes [1].

A bidirectional relationship between chronic liver diseases (CLDs) and T2DM has been previously reported, suggesting that around 70% of patients with T2DM have CLDs, along with conditions such as nonalcoholic fatty liver disease (NAFLD) [3,4]. Furthermore, patients with T2DM with CLDs, such as NAFLD, have nearly 2.2 times the risk of all-cause mortality, compared to those without these conditions [4].

Patients with T2DM who are male, older, have a higher body mass index (BMI), comorbid hypertension, a smoking habit, microalbuminuria, and lower glomerular filtration rates are at an increased risk of developing CLDs [3].

The liver plays a crucial role in glucose metabolism, maintaining blood glucose levels through the processes of glycogenolysis and gluconeogenesis. Impaired liver function, resulting from beta-cell dysfunction coupled with decreased insulin secretion, leads to impaired blood glucose regulation. As a result, glucose intolerance is observed in 60%-80% of patients with liver dysfunction [5,6]. Antidiabetic medications administered to patients with T2DM are predominantly metabolized in the liver, which is reduced or impaired in patients with CLD due to injured or extinct liver parenchyma. This increases the risk of hypoglycemic events in patients with T2DM who are on antidiabetic medications [6]. Even in the absence of T2DM, hypoglycemia is known to be a crucial prognostic factor in patients with CLDs [5]. Therefore, treatment strategies aimed at improving glycemic control in patients with T2DM with CLDs, while avoiding hypoglycemic events, are essential.

Currently, there are no guidelines for the optimal insulin formulation to manage patients with T2DM with CLDs [6]. However, long-acting insulin analogs, such as glargine, detemir, and degludec, are commonly prescribed for initial treatment due to their stability, persistent effects, longer duration of action, and reduced risk of glucose fluctuations and hypoglycemia [6]. Among these analogs, insulin degludec is notable for its ultra-long duration of activity, which exceeds 42 hours [7]. Additionally, degludec is notable for its low variability both within a single day and between days [8].

A noninferiority trial comparing insulin degludec and glargine has already established the safety and efficacy of the former [9]. Additionally, studies have reported a lower incidence of hypoglycemic events with insulin degludec compared to other insulin analogs, such as glargine and detemir [10].

Furthermore, patients with diabetes spend an average of 9600 USD per year on medical treatment, while in India, patients with diabetes with complications incur an average of 30,000 INR per year in medical expenses [11]. Insulin degludec has been reported as a cost-effective alternative to insulin glargine in low- and middle-income countries [12,13].

Despite its notable efficacy, reduced hypoglycemic adverse events, and cost-effectiveness, only a single study conducted in 2014 has assessed the use of insulin degludec in patients with liver dysfunction [14], emphasizing the need for further research on its safety and efficacy in patients with T2DM with comorbid CLD. Therefore, the primary objective of this study was to evaluate the effectiveness of insulin degludec in managing T2DM in patients with CLD in India based on changes in glycated hemoglobin (HbA1c), fasting plasma glucose, and postprandial glucose (PPG) levels, and the secondary objective was to determine its safety, based on the incidence of hypoglycemic events.

## Materials and methods

Study design and population

This was a single-center, retrospective, real-world observational study conducted at a cardio-diabetic center in West Delhi, India. This study included data from patients who visited the center between January and December 2023. As this was a retrospective analysis, ethical approval was not taken. The study was conducted in accordance with the principles outlined in the Declaration of Helsinki.

Eligibility criteria

Data from patients with T2DM, aged between 18 and 75 years, with a BMI of <40 kg/m2 and stable hepatic impairment classified as mild, moderate, or severe based on the Child-Pugh classification (A, B, and C, respectively) [15] were included in the analysis. Patients with advanced liver failure, hepatic encephalopathy, type 1 diabetes mellitus, a BMI >40 kg/m2, and those administered other basal insulin analogs, such as detemir and glargine, were excluded from the study.

Data collection

Electronic medical records (EMR) were used to obtain data, including demographic information such as age, gender, weight, BMI, laboratory parameters, and medical history, including the duration of T2DM, levels of fasting plasma glucose (FPG), postprandial glucose (PPG), glycated hemoglobin (HbA1c), serum albumin, bilirubin, prothrombin time, and the presence of ascites which was determined using ultrasound.

Data on baseline antidiabetic medications, baseline degludec dose, and the severity of CLD based on Child-Pugh scores were also collected. Patients with good hepatic function (Child-Pugh scores=5-6) were classified as class A, those with moderately impaired hepatic function (Child-Pugh scores=7-9) as class B, and those with advanced hepatic dysfunction (Child-Pugh score=10-15 points) as class C [15].

The FPG, PPG, HbA1c levels, Child-Pugh scores, and the dose of insulin degludec at baseline and three months after initiating degludec treatment were obtained from the EMR. The standard criteria for the laboratory parameters were: HbA1c 5.8%-6.3%; FPG 60-110 mg/dL; PPG 100-140 mg/dL. The insulin dose titration was done depending on the blood glucose levels, as decided by the physician, to bring FPG levels close to 100 mg/dL. The data on the incidence of hypoglycemia at the end of the three months was also obtained from the records. Incidence of nocturnal hypoglycemia, which was noted by the patients using a glucometer were also obtained from the records.

Endpoints

The effectiveness of insulin degludec was assessed by changes in FPG, PPG, and HbA1c levels from baseline to the end of the three-month degludec treatment period. The safety of degludec was evaluated by the incidence of level 1, 2, 3, or nocturnal hypoglycemia: level 1 was defined as blood glucose levels <70 mg/dL to ≥54 mg/dL; level 2 as blood glucose levels <54 mg/dL with neuroglycopenic symptoms requiring immediate action; [16] and level 3 as a severe event with altered physical and/or mental status, requiring emergency assistance for recovery and potentially progressing to loss of consciousness, seizure, coma, or even death [16]. Changes in the dose of insulin degludec and Child-Pugh scores were also noted.

Statistical analysis

Descriptive statistics were used to present the data; categorical data are represented as frequencies and percentages, and continuous data as mean and standard deviation (SD). The change in efficacy parameters from baseline to the end of the three-month treatment with insulin degludec was analyzed using the nonparametric Wilcoxon signed-rank test due to the small sample size. A p-value of <0.05 was considered statistically significant. The data were analyzed using R software, version 4.3.2 (Foundation for Statistical Computing, Vienna, Austria). Additionally, an exploratory analysis was performed using pairwise comparison to assess whether factors such as baseline antidiabetic medications and Child-Pugh scores influenced the outcome parameters.

Further, a post-hoc power analysis was conducted for the paired outcomes (FPG, PPG, and HbA1c), using paired t-tests. The effect sizes (Cohen’s d) observed for the three outcome variables were 2.08 for FPG, 1.63 for PPG, and 2.24 for HbA1c. With a sample of 35, the calculated statistical power for each test was greater than 99% (power=1.0), which was above the conventional threshold of 80% required to detect clinically meaningful differences. These findings confirm that despite the modest sample size, the study was sufficiently powered to detect the treatment effects.

## Results

A total of 35 patients were enrolled in the study, of whom 25 (71.4%) were male, with a mean age of 59.43 years (SD: 11.51). Most of the patients (20, 57.2%) enrolled in the study had been living with T2DM for fewer than 10 years. Most of the patients (19, 54.3%) were categorized as class A based on Child-Pugh scores, indicating mild liver impairment. The baseline demographics of the patients are presented in Table 1.

**Table 1:** Baseline demographics of patients with T2DM. BMI: body mass index; T2DM: type 2 diabetes mellitus; N: number; %: percentage; SD: standard deviation.

Variable	Mean (SD)
Age	59.43 (11.51)
BMI	27.05 (2.51)
Weight	71.12 (8.73)
Duration of T2DM, N (%)	
0–5 years	10 (28.6)
6–10 years	10 (28.6)
11–15 years	8 (22.9)
16–20 years	6 (17.1)
21–25 years	1 (2.9)
Child–Pugh classification, N (%)	
Class A (score: 5–6)	25 (71.4)
Class B (score: 7–9)	10 (28.6)
Class C (score: 10–15)	0 (0)

The distribution of T2DM treatments at baseline is outlined in Table 2. All patients (100%) were on insulin, while nearly half (48.6%) were also on sulfonylureas, and others were on metformin (37.1%), dipeptidyl peptidase-4 inhibitor (34.3%), and sodium-glucose transport protein 2 inhibitors (20.0%) at baseline.

**Table 2:** Distribution of baseline T2DM treatments. *Patients were on more than one medication for diabetes. DPP4i: dipeptidyl peptidase 4 inhibitor; SGLT2i: sodium–glucose transport protein 2 inhibitor; T2DM: type 2 diabetes mellitus; N: number of patients; %: proportion of patients.

	Subcategories	N (%)*
Baseline T2DM treatment	Sulfonylureas	17 (48.6
	Metformin	13 (37.1)
	DPP4i	12 (34.3)
	SGLT2i	7 (20.0)
	Insulin	35 (100)

**Figure 1:**
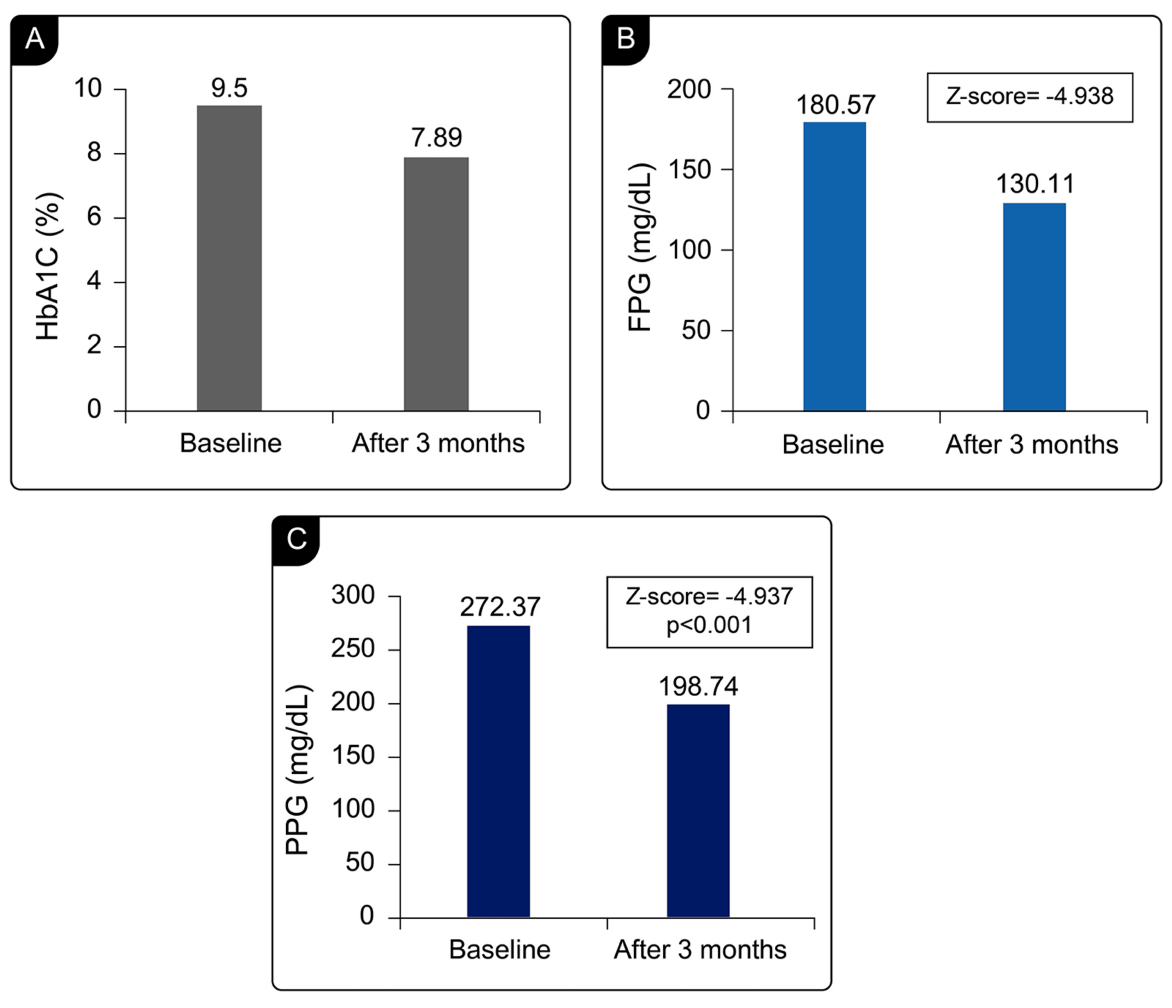
Comparison of glycemic variables at baseline and end of the study: A) HbA1c levels, B) FPG, and C) PPG levels. FPG: fasting plasma glucose; HbA1c: glycated hemoglobin; PPG: postprandial glucose.

The changes in glycemic parameters from baseline to three months after degludec treatment are presented in Figure 1. Significant reductions were observed in glycemic parameters, such as HbA1c (baseline=9.50±1.30%; at three months=7.89±1.03%; absolute change=1.61%; Z-score=-5.090, p<0.001), FPG (baseline=180.57±30.30 mg/dL; at three months=130.11±18.64 mg/dL; absolute change=50.46mg/dL; Z-score=-4.938, p<0.001), and PPG (baseline=272.37±48.10 mg/dL; at three months=198.74±22.98 mg/dL; absolute change=73.63 mg/dL; Z-score=-4.937, p<0.001). Significant changes in liver function parameters, such as bilirubin levels (baseline=1.29±0.57 mg/dL; at three months=0.99±0.47 mg/dL; absolute change=0.3%; Z-score=-4.002, p<0.001), AST (baseline=54.43±85.42 U/L; at three months=47.66±65.28 U/L; Z-score=-3.106, p=0.002), ALT (baseline=62.19±98.33 U/L; at three months=49.13±55.06 U/L; Z-score=-2.793, p=0.005), and prothrombin time (baseline=1.40±0.25s; at three months=1.31±0.19s; absolute change=0.09s%; Z-score=-4.056, p<0.001), were also observed from baseline to the endpoint after three months.

The mean (SD) dose of insulin degludec increased from 10.40 (1.59) to 17.37 (3.35) units over the three months (Z-score=-5.195, p<0.001). The univariate analysis of variance (ANOVA) results for each difference outcome showed no statistically significant effects of baseline antidiabetic medications, Child-Pugh scores, or their interaction on changes in FPG, PPG, or HbA1c levels. Therefore, the change in outcome parameters observed may be attributed to the use of insulin degludec (Table 3).

**Table 3:** Influence of baseline antidiabetic medications and Child Pugh scores on change in primary outcome parameters. Df: degrees of freedom; FPG: fasting plasma glucose; HbA1c: glycated hemoglobin; PPG: postprandial glucose.

Outcome parameters	Df	Sum Sq	Mean Sq	F-value	p-value
Change in FPG					
Baseline antidiabetic medications	2	405	202.4	0.284	0.755
Child Pugh scores	1	97	97.4	0.137	0.714
Baseline antidiabetic medications:Child Pugh scores	2	709	354.5	0.498	0.613
Residuals	26	18512	712		
Change in PPG					
Baseline antidiabetic medications	2	1191	595.3	0.256	0.776
Child Pugh scores	1	879	878.8	0.377	0.544
Baseline antidiabetic medications:Child Pugh scores	2	5150	2575.2	1.105	0.346
Residuals	26	60574	2329.8		
Change in HbA_1c_					
Baseline antidiabetic medications	2	2.52	1.2581	0.822	0.451
Child Pugh scores	1	0.15	0.1508	0.098	0.756
Baseline antidiabetic medications:Child Pugh scores	2	0.57	0.2872	0.188	0.830
Residuals	26	39.82	1.5315		

**Figure 2:**
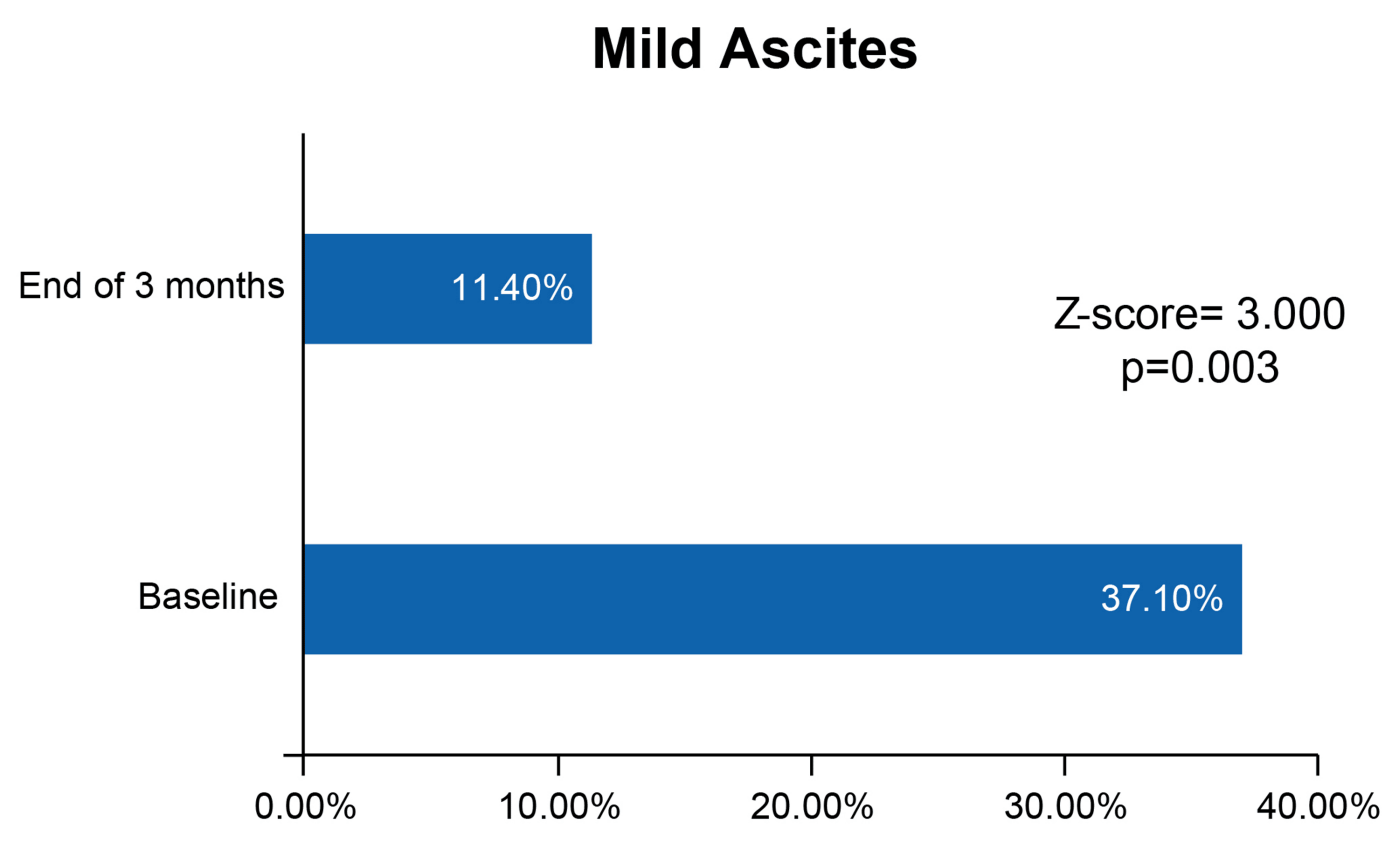
Reduction in mild ascites from baseline to three months after treatment.

Among the adverse events, a total of 10 patients reported hypoglycemia: five patients (14.3%) developed level 1 hypoglycemia, and five (14.3%) patients developed nocturnal hypoglycemia. There were no reports of level 2 or 3 hypoglycemia. Furthermore, it was observed that during the study period, liver function improved, and the proportion of patients with mild ascites decreased significantly by the end of the study (baseline=37.1%; at three months=11.4%; Z-score=3.000, p=0.003) (Figure 2).

## Discussion

Data on the use of insulin degludec in patients with T2DM with CLD in India are limited. This retrospective study, therefore, assessed the safety and effectiveness of insulin degludec in patients with T2DM with CLD. The study findings suggest that insulin degludec could be considered a safe and effective alternative for managing patients with T2DM with CLD. Significant improvements in glycemic parameters, such as FPG, PPG, and HbA1c, from baseline to the end of three months, indicate the effectiveness of insulin degludec in improving glycemic control.

CLD in patients with T2DM is a dual insult to liver function, resulting in a higher risk of acute decompensation events, including ascites, hepatic encephalopathy, and bacterial infections, along with an increased risk of morbidity and mortality [17]. In patients with T2DM, CLD usually manifests as NAFLD, with or without hepatocellular carcinoma or liver failure, necessitating a liver transplant [17]. Furthermore, achieving glycemic control in patients with T2DM with CLD is challenging due to the risk of hypoglycemia and the possibility of altered drug metabolism, among other factors, such as the risk of lactic acidosis, malnutrition, and sarcopenia [18].

In this study, insulin degludec, an ultra-long-acting basal insulin analog, helped achieve glycemic control in patients with T2DM with CLD. The ultra-long-lasting effect and stable pharmacokinetics of insulin degludec were previously reported in a study involving patients with varying degrees of hepatic impairment [14].

Lower HbA1c, FPG, and PPG levels were observed after three months of insulin degludec treatment in the current study. A recent real-world Tresiba Real-World Use Study (TRUST) registry, conducted in India, assessed the long-term safety and effectiveness of insulin degludec and reported similar improvements in glycemic parameters among adult patients with diabetes mellitus [19]. A mean dose of 0.4 U/kg was reported by Kupčová et al. to achieve steady-state insulin exposure over a 24-hour dosing interval, regardless of the severity of hepatic impairment [14]. Considering the mean body weight of 71 kg among patients in the present study, the increase in the dose of insulin degludec to 17 U at the end of three months seems justified for achieving optimum insulin exposure.

Improvements in liver function in terms of bilirubin levels were observed in this study, along with a reduction in AST and ALT. However, studies with larger sample sizes should be conducted to evaluate the effectiveness of insulin degludec in patients with moderate-to-severe CLDs.

In this study, apart from the effectiveness of insulin degludec, the incidence of adverse events in the form of hypoglycemia was very low, with none of the patients reporting moderate-to-severe hypoglycemia. This was consistent with Kupčová et al.’s study, which reported two episodes of mild hypoglycemia in a single patient with T2DM [14]. Insulin degludec has also been previously reported as a safer alternative, with a lower incidence of hypoglycemia, in comparison with other long-acting basal insulin analogs [6,10]. The BEGIN trials conducted in patients with T2DM reported a reduced incidence of nocturnal hypoglycemia with degludec compared to glargine [20]. Similarly, the DEVOTE trial reported a decreased incidence of severe hypoglycemic events with degludec, when compared to glargine, in patients with T2DM and comorbid cardiovascular diseases, kidney dysfunctions, or both [21]. This may also suggest the effectiveness of insulin degludec across a broader population of patients with T2DM and diverse comorbidities, along with a lower risk of adverse effects.

Limitations

Despite being one of the few studies published on the effectiveness and safety of insulin degludec in patients with T2DM with CLD, this study has a few limitations. The major limitation of the study is the lack of a comparator arm that limits the direct estimate of the relative safety and effectiveness of insulin degludec. Further limitations include the retrospective design, which may restrict causal inference, and a smaller sample size that can reduce the generalizability of the study findings. Additionally, the generalizability of the findings could be further limited by the single-center study design and potential bias in patient selection. Larger prospective randomized controlled trials with a comparator arm must be conducted in patients with T2DM with advanced CLD (based on Child-Pugh scores), as the present study predominantly included patients with mild to moderate CLD (Child-Pugh class A and B) but none with severe CLD (Child-Pugh class C), to further confirm the safety and efficacy of degludec. Further, the study duration was three months, which was considered sufficient to determine the preliminary clinical efficacy of insulin degludec in patients with CLD, as dose titration, stabilization, and hypoglycemic events occur during this period. However, long-term follow-up studies are necessary to determine the long-term efficacy of insulin degludec in this cohort of patients.

## Conclusions

This retrospective study on the effectiveness of insulin degludec in managing T2DM in patients with CLD showed an improvement in glycemic parameters, such as reductions in HbA1c, FPG, and PPG levels. In addition, the incidence of adverse events, such as hypoglycemia, was found to be lower. These preliminary findings suggests that degludec can be considered a safe and effective alternative for these patient cohorts. However, the short study duration, small sample size, and the lack of a comparator arm may limit the generalizability of the study. Further prospective research is warranted with a larger sample size and a comparator arm in patients with T2DM with advanced CLD.
